# The *Pseudomonas aeruginosa* PSL Polysaccharide Is a Social but Noncheatable Trait in Biofilms

**DOI:** 10.1128/mBio.00374-17

**Published:** 2017-06-20

**Authors:** Yasuhiko Irie, Aled E. L. Roberts, Kasper N. Kragh, Vernita D. Gordon, Jaime Hutchison, Rosalind J. Allen, Gavin Melaugh, Thomas Bjarnsholt, Stuart A. West, Stephen P. Diggle

**Affiliations:** a School of Life Sciences, Centre for Biomolecular Sciences, University of Nottingham, Nottingham, United Kingdom; b Department of Biology and Biochemistry, University of Bath, Bath, United Kingdom; c Department of Biology, University of Dayton, Dayton, Ohio, USA; d Department of International Health, Immunology and Microbiology, University of Copenhagen, Copenhagen, Denmark; e Center for Nonlinear Dynamics and Department of Physics, The University of Texas at Austin, Austin, Texas, USA; f School of Physics and Astronomy, University of Edinburgh, Edinburgh, United Kingdom; g Department of Clinical Microbiology, Rigshospitalet, Copenhagen, Denmark; h Department of Zoology, University of Oxford, Oxford, United Kingdom; University of Oxford; California Institute of Technology/HHMI

**Keywords:** *Pseudomonas aeruginosa*, social evolution, biofilms

## Abstract

Extracellular polysaccharides are compounds secreted by microorganisms into the surrounding environment, and they are important for surface attachment and maintaining structural integrity within biofilms. The social nature of many extracellular polysaccharides remains unclear, and it has been suggested that they could function as either cooperative public goods or as traits that provide a competitive advantage. Here, we empirically tested the cooperative nature of the PSL polysaccharide, which is crucial for the formation of biofilms in *Pseudomonas aeruginosa*. We show that (i) PSL is not metabolically costly to produce; (ii) PSL provides population-level benefits in biofilms, for both growth and antibiotic tolerance; (iii) the benefits of PSL production are social and are shared with other cells; (iv) the benefits of PSL production appear to be preferentially directed toward cells which produce PSL; (v) cells which do not produce PSL are unable to successfully exploit cells which produce PSL. Taken together, this suggests that PSL is a social but relatively nonexploitable trait and that growth within biofilms selects for PSL-producing strains, even when multiple strains are on a patch (low relatedness at the patch level).

## IMPORTANCE

Many studies have shown that bacterial traits, such as siderophores and quorum sensing, are social in nature. This has led to an impression that secreted traits act as public goods, which are costly to produce but benefit both the producing cell and its surrounding neighbors. Theories and subsequent experiments have shown that such traits are exploitable by asocial cheats, but we show here that this does not always hold true. We demonstrate that the *Pseudomonas aeruginosa* exopolysaccharide PSL provides social benefits to populations but that it is nonexploitable, because most of the fitness benefits accrue to PSL-producing cells. Our work builds on an increasing body of work showing that secreted traits can have both private and public benefits to cells.

## INTRODUCTION

The growth and proliferative success of many bacteria, including human pathogens, depend upon their ability to form biofilms in their respective environmental niches. Biofilms are multicellular three-dimensional structures that are held together by extracellular matrix molecules that encapsulate cells and cause them to aggregate. These extracellular polysaccharides (EPS) that are secreted by the bacteria typically function as adhesins, which are used to attach cells to a surface and to maintain the three-dimensional biofilm structure, and sometimes aid in protection against a variety of stresses, including dehydration, antibiotics, and predators (1, 2). The production of EPS represents a problem from an evolutionary perspective (3), because it appears to be a type of cooperative behavior that can potentially provide a benefit to all cells in the community (i.e., a “public good”) and not just to those that produce EPS. Consequently, the question arises: “what prevents the invasion of potential ‘cheats’ that do not produce EPS?” (4). Such cheats would presumably have a fitness advantage, as they could gain the benefits of EPS produced by others without paying the metabolic cost of EPS production.

Two types of hypotheses have been suggested to explain why the costly production of EPS may be evolutionarily stable. The first hypothesis assumes that the production of EPS provides a benefit to the local population of cells, which can be exploited by cells not producing EPS (5, 6). This is directly analogous to a range of public goods that have been studied in bacteria, such as iron-scavenging siderophore molecules and quorum sensing (QS) (7, 8). Both theories and experiments have shown that the production of exploitable public goods is favored in spatially structured populations, which leads to a high relatedness between interacting cells, such that EPS producers tend to be aggregated and cooperate with other EPS producers (7–9). The second possibility is that the production of EPS does not help biofilm growth *per se*, but it helps the EPS-producing lineage to outcompete other lineages with which they are interacting. For example, EPS-producing cells might be able to spatially smother or displace nonproducing lineages (10, 11). In this case, EPS is not an exploitable (cooperative) public good; instead, it is a trait that provides an advantage in competition for resources.

From an evolutionary perspective, these two hypotheses are different. The first involves a cooperative (or “prosocial”) trait that provides a benefit at the group level, whereas the second involves a trait that is costly at the group level but provides a competitive advantage to a lineage. Consequently, although both traits are social, they are different types of social traits (12, 13). The different assumptions behind these hypotheses lead to different predictions. If EPS molecules are public goods, then monocultures of EPS producers will grow biofilms faster than nonproducers, yet the nonproducers will be able to outcompete the producers in mixed biofilms (producers will have increased absolute growth and decreased relative growth) (5, 9). In contrast, if the function of EPS is to provide a competitive advantage, then we arrive at the opposite prediction, that the nonproducers will grow faster in monoculture and the EPS producers will grow faster in mixed biofilms (producers will have decreased absolute growth but increased relative growth) (11). Support for these different hypotheses has already been demonstrated by examining different EPS in different species—as a public good in *Pseudomonas fluorescens* and *Bacillus subtilis* (6, 14) and as a competitive advantage in *P. fluorescens*, *Pseudomonas aeruginosa*, and *Vibrio cholerae* (15–18).

EPS molecules produced by different bacterial species can vary greatly in both their chemical structure and the biological roles they play within biofilms (19). EPS can even have consequences for other social traits, such as facilitating the transfer of other public goods (20). Furthermore, many species produce more than one type of EPS, and these are sometimes, but not necessarily, coregulated. This means that there could be considerable variation in the social nature of different types of EPS and for different bacterial species.

Here, we examine the social nature of an EPS molecule produced by *P. aeruginosa*, an opportunistic pathogen that causes various biofilm infections, such as chronic respiratory infections of cystic fibrosis (CF), keratitis, and chronic wound infections. It produces at least three different types of EPS molecules as major components of its biofilm matrix: alginate, PEL, and PSL polysaccharides (21, 22). Alginate and PSL production are inversely regulated (23, 24), and alginate is not expressed at high levels in the majority of non-CF isolates (25, 26). In contrast, PSL is expressed by most *P. aeruginosa* natural and clinical isolates (25). PSL is a crucial adhesive scaffolding component of the biofilm matrix, promoting both cell-cell interactions and surface attachment (27–29). PSL also has a unique function as an intercellular signaling molecule (30), underscoring its roles in social evolutionary interactions. We found that PSL production is a social trait that provides benefits at the individual and group levels, but it cannot be successfully cheated by individuals who do not produce it. Our results therefore point to a scenario that is distinct from either of the hypotheses that have been proposed. Our work adds to the increasing body of work that shows that not all components of the biofilm matrix act as shared resources.

## RESULTS

### PSL provides a population-level benefit in biofilms.

We first tested whether PSL provides fitness benefits to *P. aeruginosa* populations growing in biofilms or nonbiofilms, compared to growth of populations of mutants that do not produce PSL. To this end, we measured the amount of biofilm and nonbiofilm biomasses produced over 4 days of growth for PSL producer and nonproducer strains. In order to simultaneously monitor both unattached and biofilm subpopulations within a microcosm, we modified a previously described bead method (31). This model allows us to grow biofilms on 7-mm plastic beads in test tubes, harvest biofilm cells from the beads, and directly aspirate cells that are not attached to beads from the liquid medium of the same culture (see Fig. S1 in the supplemental material). Cells not attached to beads likely include all (or combinations of any) of the following: unattached multicellular aggregates, free-swimming planktonic cells, cells defective in biofilm formation, and cells dispersed from biofilms. Since these cell types can all be transient and inseparable or indistinguishable, in this paper we unify all of these types of cells and refer to this subpopulation as “unattached cells.” We used two strains: a PSL^+^ strain that constitutively produces PSL and a PSL^−^ mutant that produces no PSL. PSL expression has been shown to induce an increase of the intracellular concentration of the secondary messenger molecule c-di-GMP, which controls multiple biofilm-associated genes (30). Consequently, to ensure that our results are due to PSL production specifically and not downstream c-di-GMP-dependent pleiotropic effects, we constitutively elevated c-di-GMP (in a Δ*wspF* mutant background) in both our strains; nevertheless, we ultimately found that our results were c-di-GMP independent, because qualitatively identical results were recorded when we used non-*wspF*-mutated backgrounds (Fig. S2). Δ*wspF* strains constitutively upregulate both *psl* and *pel* transcription (32), thus maximizing the phenotypic effects of the EPS in question. It is noteworthy that Δ*wspF* mutants are frequently selected for in *in vitro* biofilms and *in vivo* biofilm-related infections (33, 34). Furthermore, to ensure our results were solely dependent on PSL, we mutated the other c-di-GMP-coregulated EPS gene locus, *pel*. Thus, in this paper, unless specified otherwise, our “PSL^+^ strain” is Δ*wspF* Δ*pel* and the “PSL^−^ strain” is Δ*wspF* Δ*pel* Δ*psl*. Later, when we address the PEL polysaccharides, the “PEL^+^ strain” is Δ*wspF* Δ*psl* and the “PEL^−^ strain” is Δ*wspF* Δ*pel* Δ*psl*.

10.1128/mBio.00374-17.1 FIG S1  Schematic of the bead biofilm system. Download FIG S1, JPG file, 0.05 MB. Copyright © 2017 Irie et al. 2017 Irie et al. This content is distributed under the terms of the Creative Commons Attribution 4.0 International license.

10.1128/mBio.00374-17.2 FIG S2  PSL-dependent social traits are independent of c-di-GMP. (A) Arabinose-induced PSL-overexpressing Δ*pel* P_BAD_-*psl* strain consistently produced more biofilm biomass than the defective Δ*pel* Δ*psl* mutant (*F*_1,16_ = 17.54, *P* = 0.0007). Conversely, no significant differences between the strains were seen in the unattached fractions of the cultures (*F*_1,15_ = 11.3, *P* > 0.4). (B) While the experiments in the figures of the main text were performed using the Δ*wspF* mutant strain background (with constitutively elevated c-di-GMP), to remove c-di-GMP and other c-di-GMP-regulated factors from playing a role in our interpretations, we repeated the experiment without mutating *wspF*, and this yielded identical results for both biofilm populations (left panel) and unattached populations (right panel) (compare with Fig. 2A), despite the Δ*pel* Δ*psl* mutant having low intracellular c-di-GMP and the Δ*pel* P_BAD_-*psl* mutant having high intracellular c-di-GMP (30). The cultures were grown in 1% l-arabinose. Download FIG S2, JPG file, 0.1 MB. Copyright © 2017 Irie et al. 2017 Irie et al. This content is distributed under the terms of the Creative Commons Attribution 4.0 International license.

**FIG 1:**
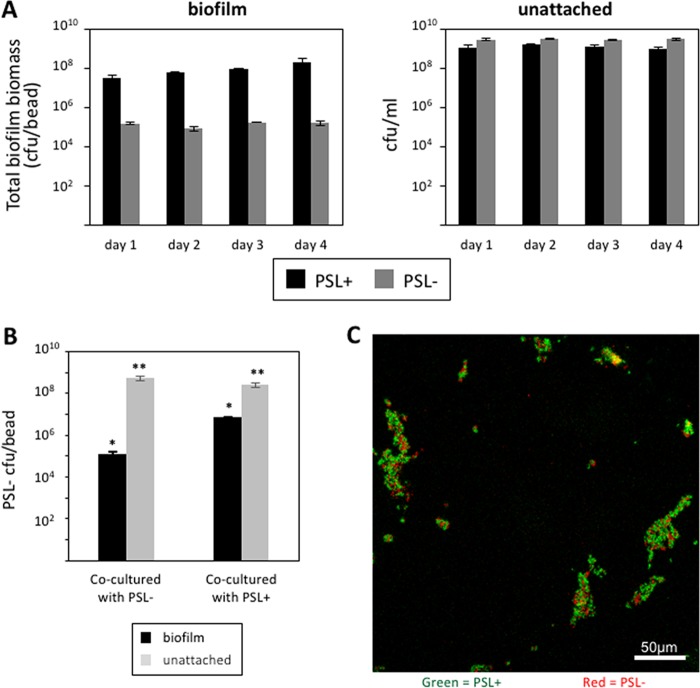
PSL production provides community benefits to cells grown in biofilms. (A) The PSL^−^ strain was significantly defective in biofilm formation on beads compared to the PSL^+^ strain (*F*_1,15_ = 11.26, *P* = 0.0043; *n* = 3), but no major differences were seen between either strain’s growth in the unattached fractions (*F*_1,16_ = 0.67, *P* = 0.4251; *n* = 3). (B) PSL^−^ cells cocultured with PSL^+^ cells at a 1:1 ratio increased their proportional numbers in a biofilm. *, *P* < 0.0001; **, *P* > 0.08; *n* = 4. (C) Confocal micrograph images of surface-attached populations of PSL^−^/PSL^+^ cocultures at a 1:1 ratio. PSL^−^ cells (red) coaggregated with PSL^+^ cells (green).

We found that PSL provides a population-level benefit in biofilms but not in unattached populations (Fig. 1A). Consistent with previous reports (25, 27–29, 35), we found that over 4 days of growth, PSL mutants formed significantly less biomass and therefore poorer biofilms than the corresponding PSL^+^ strain. In contrast, we found no significant differences in the final population densities between the PSL^+^ and the PSL^−^ strains in the unattached population of cells.

In order to confirm that our unattached population results were not biased by cells that had detached from biofilms, we also performed an experiment where we added no beads and therefore the analyses were all performed on purely unattached cells. Again, we found no significant differences in the final population densities between PSL^+^ and PSL^−^ strains (Fig. S3). Since we found that a significant biomass was achieved after 1 day of biofilm growth, and to minimize the occurrence of rapid spontaneous mutations that can develop and accumulate in mature biofilms of *P. aeruginosa* (35), we carried out all subsequent experiments with 24-h cultures.

10.1128/mBio.00374-17.3 FIG S3  EPS mutant strains show no observable differences in growth over 4 days in beadless culture tubes. Download FIG S3, JPG file, 0.1 MB. Copyright © 2017 Irie et al. 2017 Irie et al. This content is distributed under the terms of the Creative Commons Attribution 4.0 International license.

### PSL provides social benefits in biofilms.

We then asked whether the production of PSL provides social benefits to other cells within a biofilm. We tested this in unattached populations and in biofilms by growing the PSL^−^ strain with either other PSL^−^ cells or PSL^+^ cells in a 1:1 ratio. We found that approximately 100-fold more PSL^−^ cells could attach and form biofilms in the presence of PSL^+^ than when PSL^−^ was cocultured with PSL^−^ (Fig. 1B). In contrast, in unattached populations, we found that the fitness of PSL^−^ cells was not influenced by coculturing with PSL^+^ (Fig. 1B). This indicated that, in biofilms, the production of PSL provides some benefits to cells that do not produce PSL, but this is not true for unattached populations. Consistent with PSL^+^ providing a benefit to PSL^−^ cells, we found that when we cocultured PSL^+^ and PSL^−^ strains, PSL^−^ cells coaggregated with PSL^+^ cells and incorporated themselves into the biofilm during the early stages of biofilm formation (Fig. 1C).

### PSL mutants do not act as social cheats within biofilms.

**FIG 2:**
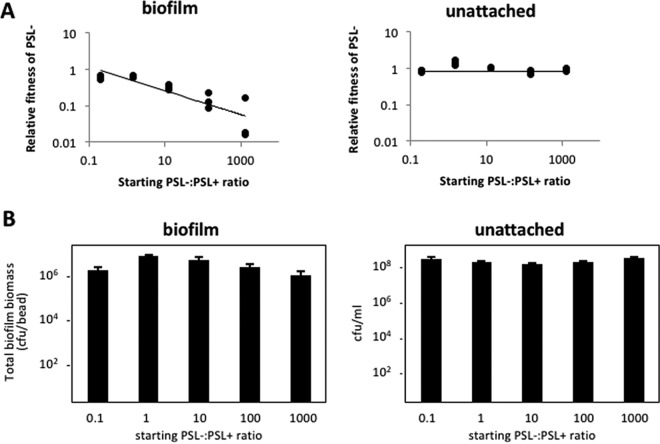
PSL mutants are not social cheats within biofilms. (A) The relative fitness of PSL^−^ cells was equal or lower than that of the PSL^+^ strain across all starting frequencies of the mutant in mixed cultures in biofilms (*t*^13^ = −3.242, *P* = 0.0064). PSL^−^ strain fitness was equal to that of the PSL^+^ strain across all starting frequencies in unattached populations (*t*^13^ = −0.741, *P* = 0.4716). (B) The PSL^−^/PSL^+^ cell coculture biomass remained similar across all starting ratios in both biofilm (*t*^13^ = −1.769, *P* = 0.1004) and unattached populations (*t*^13^ = 1.881, *P* = 0.0825).

We next tested whether PSL^−^ strains could act as social cheats (4), i.e., whether these strains increased in frequency when growing in mixed cultures with the PSL^+^ strain. We varied the starting PSL^−^:PSL^+^ cell ratios from 0.1 to 10,000, because theory predicts that the fitness of cheats should be frequency dependent, with cheats being better able to exploit cooperators when the cheats are rarer (36). In biofilms, we found that the relative fitness of the PSL^−^ cells was either equal to or lower than that of the PSL^+^ strain, suggesting that the PSL^−^ cells were not able to outcompete the PSL^+^ cells (i.e., to cheat). The relative fitness of the PSL^−^ strain was negatively correlated with its starting frequency in the population (Fig. 2A). At high starting frequencies of PSL^+^ cells, the relative fitness of the PSL^−^ strain was not significantly different from that of the PSL^+^ strain. As the starting frequency of the PSL^−^ strain was increased, the relative fitness of the PSL^−^ strain became lower than that of the PSL^+^ strain. Thus, the PSL^−^ strain does not outcompete the PSL^+^ strain at any starting frequency, and at a high starting frequency it is in fact outcompeted by the PSL^+^ strain. In contrast, we found that in unattached populations, the relative fitness of the PSL^−^ strain did not differ significantly from the PSL^+^ strain, irrespective of the starting ratio (Fig. 2A).

**FIG 3:**
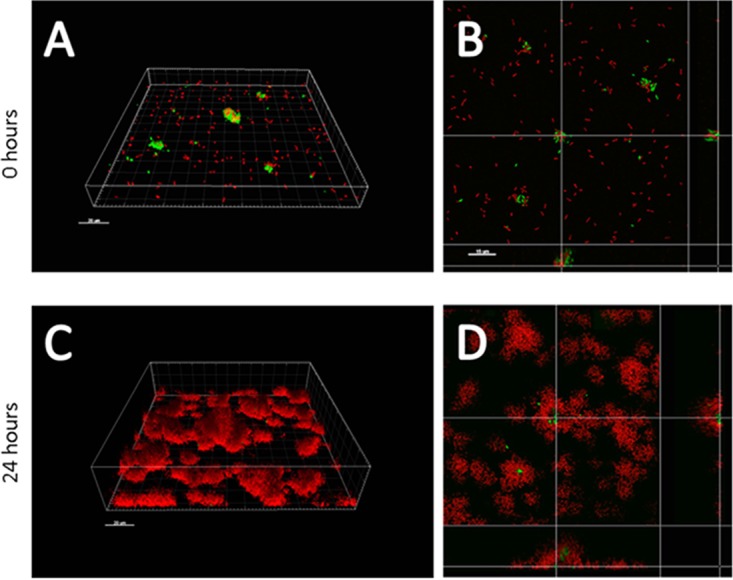
The PSL^+^ strain smothers and outcompetes the PSL^−^ strain during biofilm growth. Confocal micrograph images of PSL^−^/PSL^+^ (1:1 cell ratio) mixed population biofilms at 0 h (A and B) and 48 h (C and D). PSL^−^ cells were only attached to the surface as coaggregates with PSL^+^ cells, and they were eventually outcompeted by PSL^+^ cells when the biofilm matured. Panels A and C show three-dimensional rendered images, and panel B and D represent corresponding open box top-down views. Red, PSL^+^ cells; green, PSL^−^ cells.

We also tested whether the presence of the PSL^−^ strain adversely affected biofilm production in mixed cultures. Given that the PSL^−^ strain is unable to form biofilms to the same biomass level as the PSL^+^ strain (Fig. 1), we expected that a high starting frequency of the PSL^−^ strain would adversely affect biofilm production. In contrast to this expectation, we found that the final biomass did not significantly change as we varied the starting PSL^−^:PSL^+^ cell ratio from 0.1 to 1,000 (Fig. 2B). This result indicated that the final biomass is determined by the PSL^+^ cells, which dominate long-term biofilm growth independently of the starting ratio between PSL^−^ and PSL^+^ cells. To allow us to microscopically visualize growth of the PSL^−^ and PSL^+^ strains in coinoculated biofilms, we performed a biofilm flow cell experiment with a mixed PSL^+^ and PSL^−^ population. Consistent with the results shown in Fig. 1C, PSL^−^ cells appeared to coaggregate with PSL^+^ cells and incorporated themselves into the early-stage biofilms (Fig. 3A and B). However, over time, the PSL^+^ cells covered and outcompeted the PSL^−^ cells (Fig. 3C and D).

### PSL provides population-level benefits against antibiotics.

**FIG 4:**
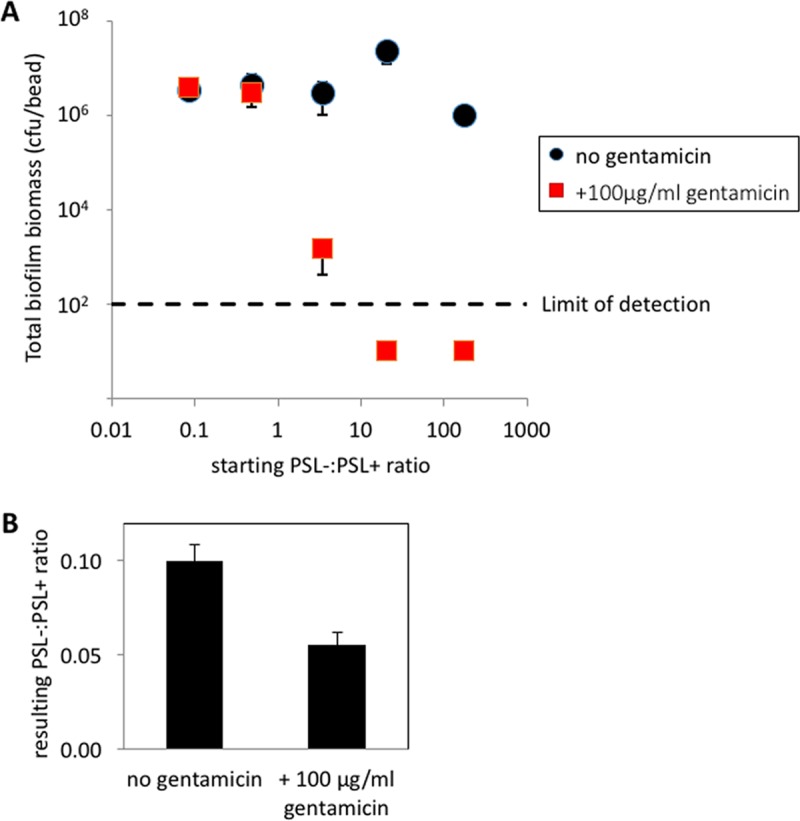
PSL production results in increased tolerance toward antibiotics. (A) PSL^−^/PSL^+^ cell mixed biofilms yielded consistent biomasses regardless of the starting ratio of the two strains (*t*^13^ = −0.732, *P* = 0.4769; *n* = 3). Treatment of the mixed biofilm with a high concentration of gentamicin eliminated the entire biofilm population when the starting proportion of PSL^−^ cells increased above that of PSL^+^. cells. (B) In the intermediate PSL^−^:PSL^+^ coculture, where the mixed biofilm was partially eradicated, there were fewer PSL^−^ cells in the surviving biofilm in the presence of gentamicin (*t*^4^ = 4.07, *P* < 0.02; *n* = 3).

PSL is known to play a role in tolerance to antibiotics, including aminoglycosides (35, 37) (Fig. S4). We tested the social consequences of this tolerance by examining the effect of adding gentamicin at a concentration previously known to affect biofilms (100 μg/ml) (38) to populations that contained various ratios of PSL^−^ mutants. In the absence of gentamicin, we found that the frequency of PSL^−^ mutants did not influence the biomass of the biofilm (Fig. 4A), consistent with the results shown above in Fig. 2B. In contrast, when we added gentamicin, the biofilm biomass negatively correlated with the fraction of PSL^−^ mutants in the starting population (Fig. 4A). When there were more PSL^−^ than PSL^+^ cells in the starting population, the addition of gentamicin led to a significant drop in viable biofilm biomass. In cases where the starting PSL^−^:PSL^+^ cell ratio was greater than 10, the addition of gentamicin reduced the population to below our threshold detection level. When we examined in more detail the populations which showed partial killing, we found that PSL^+^ cells had a significantly higher level of survival than the PSL^−^ cells (Fig. 4B).

10.1128/mBio.00374-17.4 FIG S4  PSL expression protects cells from a bactericidal concentration of gentamicin (100 μg/ml). Gentamicin was added directly to 24-h bead biofilm cultures and incubated for an additional 24 h at 37°C with shaking at 200 rpm. Resulting populations from both biofilm and unattached cells were tested for viability by serial dilution and plating. ND, not detected. Download FIG S4, JPG file, 0.1 MB. Copyright © 2017 Irie et al. 2017 Irie et al. This content is distributed under the terms of the Creative Commons Attribution 4.0 International license.

### Strain diversity does not influence selection for PSL.

We complemented the above fitness assays with a multigenerational selection experiment. Several previous studies on microbial social traits, such as siderophore production and quorum sensing, have shown that the relative fitness of individuals that do and do not perform social traits depends upon population structure (7, 8, 39, 40). Specifically, structured populations, with a relatively low strain diversity per patch (high relatedness between cells in a patch), favors genotypes that support cooperative traits. Conversely, populations with relatively high strain diversity (low relatedness) favor less cooperative cheating genotypes. These results are what is predicted by social evolution theory (13, 41). In contrast, our observation that PSL^−^ cells are not able to cheat PSL^+^ cells in mixed cultures suggests that relatedness at the level of the culture might not matter for the evolution of PSL in the same way.

We tested this hypothesis with a selection experiment over 5 days, in which we started with a mixed population of PSL^+^ and PSL^−^ cells and manipulated both relatedness (low/high) and whether cells were grown in biofilms (on beads) or in cultures with no beads (with the entire population unattached) (Fig. S5). In each round of growth, we subdivided the population into six subpopulations (tubes). We varied relatedness by initiating each subpopulation with either a single clone, to give relatively high relatedness, or with multiple clones, to give relatively low relatedness. Consequently, for the high-relatedness treatment, the PSL^+^ and PSL^−^ cells became segregated into separate populations, while the low-relatedness treatment kept them mixed together in the same population. High and low relatedness therefore refers to relatedness at the level of the subpopulation/tube. If social interactions take place over a different time scale, then relatedness for that interaction will be different (42, 43); we return to this issue in the Discussion. We started our experiments with a 1:1 PSL^−^:PSL^+^ cell ratio and carried out six rounds of growth.

10.1128/mBio.00374-17.5 FIG S5  Schematic of the relatedness experiment. Tubes (populations) were initially inoculated with either single or multiple colonies (corresponding to populations that had relatively high relatedness [Hr] and relatively low relatedness [Lr], respectively) consisting of PSL^+^ cells, PSL^−^ cells, or a 1:1 mixture of both. Populations were grown separately in the presence/absence of plastic beads and pooled before the ratio of PSL^+^/PSL^−^ cells was determined. Single (Hr) or multiple (Lr) colonies were selected for progression into the next round of the experiment. If one subpopulation (PSL^+^ or PSL^−^) had increased fitness, the ratio and thus their likelihood to progress into subsequent rounds of selection increased. Download FIG S5, JPG file, 0.2 MB. Copyright © 2017 Irie et al. 2017 Irie et al. This content is distributed under the terms of the Creative Commons Attribution 4.0 International license.

**FIG 5:**
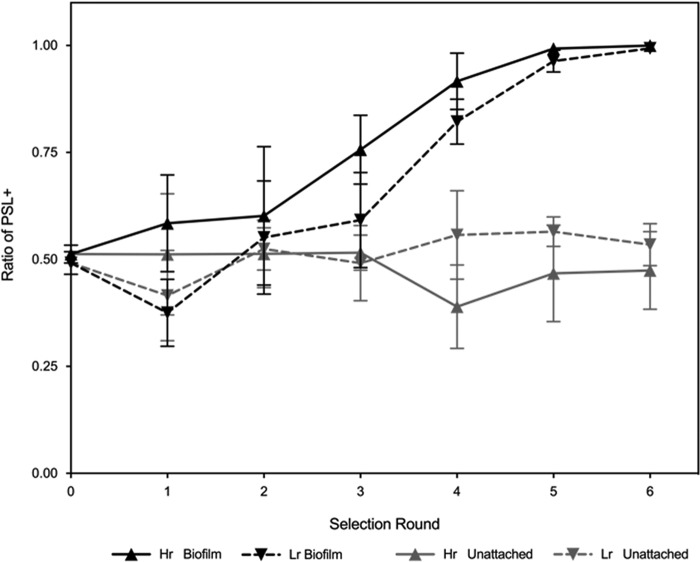
PSL production is favored in high- and low-relatedness treatments in biofilms. PSL^+^ cells had a selective advantage under conditions promoting biofilm formation (black) (*F*_1,20_ =542.7, *P* < 0.0001; *n* = 6). However, this observation was not reciprocated under unattached conditions (gray). The concurrent loss of PSL^−^ cells from both low-related and high-related biofilm populations through subsequent selection rounds suggested there is a strong selection toward PSL^+^ strains (under biofilm-promoting conditions only), as PSL^−^ strains were unable to cheat on PSL^+^ strains.

We found that, in contrast to previous selection experiments on microbial social traits, relatedness at the level of the patch had no influence on the outcome of our selection experiment (Fig. 5). Instead, we found that the relative fitness of the PSL^−^ and PSL^+^ strains was determined by whether the subpopulations were grown as biofilms or in unattached populations. Specifically, the PSL^+^ strain was significantly favored in biofilms but not in unattached populations in beadless cultures (Fig. 5). Consequently, our selection experiment provided further support for our conclusion that PSL^+^ cells are favored in biofilms and that PSL^−^ cells are unable to cheat PSL^+^ cells during growth in biofilms.

## DISCUSSION

We examined the social nature of the PSL polysaccharide produced by *P. aeruginosa* and found that (i) PSL production provides a benefit at the group level to cells growing in biofilms (Fig. 1A); (ii) PSL production provides no benefit or cost to cells growing in unattached populations (Fig. 1A and 2A); (iii) PSL^−^ cells are able to grow better as biofilms when in a mixed culture with PSL^+^ cells (Fig. 1B); (iv) PSL^+^ strains are able to outcompete PSL^−^ strains when in mixed culture biofilms, such that PSL^−^ strains cannot successfully exploit (cheat) the PSL production of the PSL^+^ strain (Fig. 2A); (v) the relative fitness of the PSL^−^ strain is lower when the PSL^+^ strain is rarer (negative frequency dependence) (Fig. 2A); (vi) the presence of PSL^−^ cells has a negligible influence on overall biofilm productivity (Fig. 2B); (vii) biofilms containing a higher proportion of PSL^+^ cells are less susceptible to antibiotics (Fig. 4A); (viii) PSL^+^ cells are better able to survive antibiotics than PSL^−^ cells when growing in a mixed culture biofilm with PSL^−^ cells (Fig. 4B); (ix) in a selection experiment, the relative success of PSL^+^ versus PSL^−^ cells was not influenced by relatedness at the patch level (Fig. 5).

Overall, our results suggest that in *P. aeruginosa* biofilms, the production of PSL is a social trait which provides benefits at the individual and group level, but that it cannot be successfully cheated by individuals who do not produce it. This is despite the fact that the PSL^−^ strain is able to gain some benefit from the presence of the PSL-producing strain in the biofilm matrix (Fig. 1). Our experiments suggest that the fitness benefits of producing PSL accrue mainly to the producing cell and/or to other PSL^+^ cells. This contrasts with previous studies on social traits in *P. aeruginosa*, where it was shown that siderophore production and QS are social traits that can be readily exploited by cheats in *in vitro* and *in vivo* biofilm experimental systems (7, 8, 40, 44). We suggest that this is because siderophores and most QS-dependent public goods are relatively freely diffusible secreted products that provide shared benefits to both producing and nonproducing cells. In contrast, the primary function of PSL is for adherence to surfaces and to other cells; PSL likely has limited diffusive properties in the biofilm biomass. PSL was previously shown to localize primarily to the periphery of biofilm microcolonies, encapsulating the cells (45). In addition, some PSL is known to be tightly associated with the cell surface (46), although some is likely to be diffusible and serve as an intercellular signaling molecule (30). This signaling property of PSL also promotes coordinated, community-level gene expression toward increased production of PSL and other biofilm-associated factors by driving its own expression in a feed-forward manner, similar to that of an autoinduction regulatory circuit.

Our results share some similarities and some differences with the two hypotheses that have been suggested to explain the evolutionary stability of EPS. Consistent with the public good hypothesis, we found that PSL production led to higher growth in monocultures and that it provided a social benefit (Fig. 1A and B and 4A) (5, 6). However, in contrast to what we would expect with a public good, we found that (i) PSL^−^ strains could not successfully exploit (cheat) the PSL production of the PSL^+^ strain (Fig. 2A); (ii) the presence of PSL^−^ cells had a negligible influence on overall biofilm productivity (Fig. 2B); and (iii) the relative success of PSL^+^ versus PSL^−^ cells was not influenced by the relatedness between cells within biofilms (Fig. 5). Consistent with the competitive advantage hypothesis, we found that PSL^+^ strains are able to outcompete PSL^−^ strains in mixed culture biofilms (Fig. 2A) (11). However, contradictory to the assumptions and predictions of the competitive advantage hypothesis, we found that (i) PSL provides an advantage, not a disadvantage, in biofilm growth, with PSL production providing a benefit at the group level to cells growing in biofilms (Fig. 1A); (ii) PSL production provides a benefit to nonproducers, with PSL^−^ strains able to grow better as biofilms when in a mixed culture with PSL^+^ cells (Fig. 1B); (iii) the benefit of producing PSL was increased by the presence of competitors (Fig. 4A).

Overall, the results of this and previous studies suggest that the social consequences of EPS can vary across species and with different types of EPS (10, 14, 16–18, 20). It is hard to disentangle whether the ability of PSL^+^ cells to outcompete PSL^−^ cells in mixed biofilms is a property of the oxygen/nutrient gradient or whether it is the property of differing adhesive strengths between PSL^+^ and PSL^−^ cells, as PSL^−^ cells are less adhesive and are more prone to be detached from the biofilm biomass under constant flow conditions when exposed on the surface. A greater adhesive ability can allow strains to displace competitors and gain an advantage in biofilms (10), as evidenced by the more evenly spaced existence of PSL^−^ cells among PSL^+^ aggregates within an early developing biofilm under no- or low-flow conditions (Fig. 1C).

Our experiments suggest that PSL production cannot be cheated, and thus it should be favored even when relatedness is low at the patch level. We tested the effect of relatedness at the patch level on selection for PSL production in biofilms in a selection experiment (Fig. 5). In our experiment, high relatedness implied that cultures were established with either PSL^+^ or PSL^−^ strains. Under these conditions, cells will interact with genetically identical cells, and PSL^−^ cells cannot interact with PSL^+^ cells. In contrast, low relatedness implies that cultures can be comprised of both PSL^+^ and PSL^−^ strains, such that PSL^−^ cells could potentially exploit the PSL^+^ cells. We found that this relatedness manipulation had no effect on our results. PSL^+^ strains were favored under conditions of high and low relatedness in biofilms but not in nonbiofilm populations. Therefore, in mixed culture biofilms, a PSL producer always wins, in contrast to previously reported findings for other social traits in *P. aeruginosa*, such as siderophore production and QS (7, 8, 40, 44). In those previous studies, relatedness at the patch level matters, because at low relatedness, the uncooperative cells are able to interact with the cooperative cells, allowing the uncooperative strains to exploit (cheat) the cooperative wild-type cells (47). A possible explanation for our results is that the benefits of PSL production are only shared locally with cells that tend to be clone mates (see above), and so the relatedness for the PSL production is always high, irrespective of the strain diversity/relatedness at the level of the culture (tube). Relatedness is a relative measure that will vary depending upon the components of the interaction, and it can therefore vary with different social traits, such as how much a product diffuses (47).

We further demonstrated that PSL helps protect biofilm communities against environmental challenges. When biofilms contain a high proportion of PSL^−^ cells, this leads to a significant increase in the susceptibility to antibiotics (Fig. 4A). Within these biofilms, PSL^+^ cells showed a higher level of survival than the PSL^−^ cells (Fig. 4B), indicating that the protection provided to the cells by PSL is most beneficial for the producing cells. This is likely due to PSL being both cell associated and released into the medium (23). While PSL^−^ cells can readily access released PSL, they do not have access to cell-associated PSL. One possibility is that there are functional differences for antibiotic tolerance provided between cell-associated PSL and released PSL. Alternatively, the relative PSL concentration may simply be higher in closer proximity to the cell surface, providing a higher level of protection against antibiotics. The shared benefit appears to be provided to the community as a whole, including nonproducing cells when they are rarer. This may be because biofilm biomass appears to be entirely encapsulated with possibly the released form of PSL (45). The exact mechanism of antibiotic tolerance by PSL is not known, and this therefore remains an open question.

Arguments have been made previously that *P. aeruginosa* cells, even when they are in the planktonic phase, have biofilm-like features (48). In our bead biofilm model, the unattached cell population, particularly those cells which constitutively express EPS, contain large numbers of cellular aggregates, as seen previously in shaken liquid cultures (30, 45). However, our results clearly distinguished the social evolutionary differences between the cells in the biofilm and unattached cells, thus providing evidence that unattached cells, whether they are aggregated or not, are definitively distinct from surface-attached biofilm cells. This raises the question whether aggregates represent a type of biofilm, are “biofilm-like,” or represent a different growth mode altogether, and our results are strongly suggestive of the latter. This is an important question, since *P. aeruginosa* biofilms growing on infected contact lenses, catheters, and other medical implants form surface-associated biofilms, but within cystic fibrosis sputum, the bacteria are thought to grow as suspended aggregates (49–51).

Most strains of *P. aeruginosa*, like many other microbial species, are capable of producing multiple biofilm EPS molecules (25, 52). Of these, PSL and PEL are coregulated by several intracellular regulatory factors, including c-di-GMP (53), and both contribute to biofilm development in a majority of *P. aeruginosa* strains, including PAO1 (25). Intriguingly, in additional experiments, we found that the social aspects of PEL production are different from those of PSL, pointing toward PEL as being a completely private and non-social good (Fig. S6). This is in contrast to recent work that inspected the competitive fitness of PEL producers using the PA14 strain, which showed community-level benefits in some circumstances (16). PA14 has a deletion mutation from the *psl* promoter through the *pslD* gene, and the nature of biofilm formation in this strain, which uses PEL polysaccharide as the sole EPS, appears to have diverged from that of other *P. aeruginosa* isolates (25). It is possible that PA14 has somehow evolved PEL to compensate for the loss of PSL by altering not only its expression patterns (52) but also its social evolutionary roles. PEL in the PAO1 strain sharply contrasts with PSL polysaccharides, or any other EPS that have been either simulated or experimentally observed (10, 11, 14, 16–18, 20). Our results and those of others highlight the importance of recognizing diverse roles and effects of different EPS as social traits within biofilms. The extracellular matrix is the material that keeps microbial cells together in a biofilm community, as it influences their social interactions and evolution. As such, we believe it holds an important foundation for our understanding of the multicellularity of unicellular organisms.

10.1128/mBio.00374-17.6 FIG S6  PEL polysaccharides are private goods. (A) Similar to PSL (Fig. 1A), the PEL^−^ strain was significantly defective in biofilm formation compared to PEL^+^ (*F*_1,24_ = 27.3, *P* < 0.0001; *n* = 4), but no major differences were seen between their growth in the unattached fractions (*F*_1,24_ = 46.56, *P* < 0.0001; *n* = 4). (B) Unlike PSL^−^ cells, cocultures of PEL^−^ cells with PEL^+^ cells did not increase the amount of PEL^−^ cells in either biofilm or unattached populations, signifying the unavailability of PEL polysaccharides to nonproducing cells. * and **, *P* > 0.02. (C) Confocal micrograph image of surface-attached populations of PEL^−^/PEL^+^ (1:1 cell ratio) cocultures. PEL^−^ cells (red) did not coaggregate with PEL^+^ cells (green). (D) There were no frequency-dependent fitness changes for PEL^−^/PEL^+^ cell cocultured biofilms (*t*^13^ = −0.802, *P* = 0.4371), and the relative fitness was consistently slightly below 1, indicating a steady disadvantage of not expressing PEL in the biofilm. There were frequency-dependent fitness changes in the unattached population (*t*^13^ = −1.435, *P* = 0.175), perhaps caused by the complex regulatory system of PEL and the involvement of quorum sensing (57). (E) Due to PEL not socially affecting coculture communities, there is a steady decline of biofilm biomass as PEL^+^ cells become rare (*F*_1,8_ = 1, *P* = 0.441099; *n* = 3), but no change was seen in the maximum cell numbers in the unattached population (*F*_1,8_ = 1.46, *P* = 0.296592; *n* = 3), regardless of the starting ratios of the strains. Download FIG S6, PDF file, 0.4 MB. Copyright © 2017 Irie et al. 2017 Irie et al. This content is distributed under the terms of the Creative Commons Attribution 4.0 International license.

## MATERIALS AND METHODS

### Bacterial strains and growth conditions.

The bacterial strains and plasmids we used and constructed for this study are listed in Table S1. We propagated *Escherichia coli* and *P. aeruginosa* strains in lysogeny broth (LB) at 37°C unless otherwise specified. The concentrations of antibiotics we used for *E. coli* were 50 μg/ml carbenicillin and 10 μg/ml gentamicin, and for *P. aeruginosa* we used 300 μg/ml carbenicillin and 100 μg/ml gentamicin. We induced P_BAD_-*psl* strains for PSL overexpression by adding 1% (wt/vol) l-arabinose. PSL is involved in a feed-forward regulation system where the expression of PSL induces the intracellular levels of c-di-GMP, which in turn overexpresses PSL (30). To avoid c-di-GMP being inadvertently triggered in our biofilm system, which would potentially affect the interpretations of our results when other c-di-GMP-induced gene products become involved, all strains used in our studies were conducted using a Δ*wspF* mutant background. Δ*wspF* mutants express biologically maximal intracellular c-di-GMP levels due to the constitutive activation of the diguanylate cyclase WspR (32).

10.1128/mBio.00374-17.7 TABLE S1  Bacterial strains and plasmids used in this study. Download TABLE S1, DOCX file, 0.03 MB. Copyright © 2017 Irie et al. 2017 Irie et al. This content is distributed under the terms of the Creative Commons Attribution 4.0 International license.

### Fluorescent strain construction.

We chromosomally labeled *P. aeruginosa* strains with either green fluorescent protein (GFP) or mCherry by using the Tn*7* delivery system (54). We electroporated 1 μl pTNS3 and 1 μl pUC18-mini-Tn7T2-PA1/04/03::*gfp* or pUC18-mini-Tn7T2-PA1/04/03::*mCherry* into a corresponding electrocompetent *P. aeruginosa* strain as previously described (55) and selected for gentamicin resistance. We removed the construct backbone by electroporating 1 μl pFLP2 and selecting for carbenicillin resistance. Upon sucrose counterselection to identify the loss of the *sacB* gene-containing pFLP2 plasmid by streaking for single colonies on LB (no salt) plus 10% sucrose at 30°C, we confirmed the strains for carbenicillin and gentamicin sensitivities.

### Bead biofilm culture system.

We grew biofilm cultures on plastic beads as previously described (31) but with several modifications (Fig. S1). We used this system because it allowed us to simultaneously study biofilm and unattached populations. This system differs from traditional flow cell models in that it is closed, and unattached populations are not lost due to flow. Therefore, any differences that we observe with our system are reflective of biofilm versus unattached populations instead of biofilm cells being the only ones left, which can occur in a flow cell system. We streaked *P. aeruginosa* strains to obtain single colonies on M9 medium with 3.062 g/liter sodium citrate (M9 citrate) agar at 37°C for 2 days. We picked individual fresh colonies and grew these to logarithmic phase (optical density at 600 nm [OD_600_, ≈0.5]) in M9 citrate broth at 37°C with shaking. We then diluted these cultures to an OD_600_ of ≈0.05 in 3 ml M9 citrate containing one or more (up to three) 7-mm plastic beads (Lascells) in standard culture tubes in independent triplicates. We then grew the cultures at 37°C with shaking at 200 rpm for 24 h. We directly aspirated unattached cells from the liquid broth portions of the cultures. We collected biofilm cells by retrieving the beads from the tubes and gently washed the beads in 10 ml phosphate-buffered saline (PBS) by five inversions, which we repeated three times. We determined the CFU of the biofilms by recovering the cells by water bath sonication in 1 ml of PBS for 10 min. We vortexed the sonicated samples for 10 s prior to serial dilution and plating.

### Antibiotic killing assays.

For antibiotic killing assays (Fig. 4), we first grew bead biofilm cultures for 24 h in the absence of gentamicin. Half the cultures were then introduced to 100 μg/ml gentamicin (final concentration), and the other half were left untreated. We then further incubated the cultures for 24 more hours at 37°C with shaking prior to harvesting the cells for analyses.

### Determining ratios of PSL^+^ and PSL^−^ cells in mixed cultures.

To distinguish between PSL^+^ and PSL^−^ cells, we introduced a single chromosomal copy of constitutively expressed GFP or mCherry genes to each strain. We then used quantitative real-time PCR and primers specifically designed against GFP or mCherry genes to accurately assess the numbers of each strain present in a sample. In order to avoid the effects of PEL, which is coregulated with PSL, interfering with or convoluting PSL-dependent phenotypes, we conducted all experiments using Δ*pel* mutant backgrounds.

### Quantitative real-time PCR.

We collected genomic DNA (gDNA) from biofilm cells from washed beads without the sonication steps by directly applying lysing reagents to the beads. We isolated unattached bacterial gDNA by pelleting 1-ml aliquots of the liquid cultures. We then stored pellets and beads at −20°C until DNA extraction. We extracted gDNA using a GenElute bacterial genomic DNA kit (Sigma) according to the manufacturer’s protocol, except for the elution buffer, which we diluted 100-fold to prevent EDTA interference with the subsequent DNA polymerase reactions. We performed real-time PCR as previously described (30) using Syber green PCR master mix (Applied Biosystems). The primers we used are listed in Table S2. We generated standard curves for *gfp* and *mCherry* genes using quantitatively determined gDNA from Δ*pel* Δ*psl* GFP strain and Δ*pel* Δ*psl* mCherry strain, respectively.

10.1128/mBio.00374-17.8 TABLE S2  Real-time PCR oligonucleotide primers used in this study. Download TABLE S2, DOCX file, 0.02 MB. Copyright © 2017 Irie et al. 2017 Irie et al. This content is distributed under the terms of the Creative Commons Attribution 4.0 International license.

### Biofilms grown on coverslips and confocal microscopy of biofilms.

Bacteria were cultured as described for the bead biofilm system, but instead of inoculating into bead-containing tubes, cultures were diluted into 1 ml M9 citrate in uncoated polystyrene 24-well plates. Each well contained one sterile 10-mm-diameter glass coverslip. Biofilms were grown in 37°C with 200 rpm shaking for 24 h. The glass coverslips were removed with forceps, gently washed three times in 10 ml PBS, and immediately mounted on a glass slide for confocal microscopy with a Zeiss LSM 700 system. Images were acquired and analyzed using Zeiss’s Zen software.

### Flow cell growth and confocal microscopy of biofilms.

We grew mixed Δ*pel* Δ*psl* and Δ*pel* mutant strain aggregates in a continuous flow cell system as described previously (56). We produced the mixed aggregates by mixing exponential-phase growing liquid cultures, adjusted to an OD_600_ of 0.001, at 1:1 in LB medium. This mix was then incubated overnight with shaking at 180 rpm. We diluted the aggregate-containing culture to an OD_600_ of 0.01 before inoculating the flow cells with 27-gauge syringes. We used a flow rate of 3 ml/h of M9 minimal medium buffered with 10% (vol/vol) A10 phosphate buffer supplemented with 0.3 mM glucose. With a Zeiss Imager Z2 microscope with an LSM 710 confocal laser scanning microscope and the accompanying Zeiss software Zen 2010 v. 6.0, we found aggregates containing a mix of the GFP- and the mCherry-tagged cells. We imaged these aggregates over time with *z*-stack intervals of 1 μm. Three-dimensional projections of images were produced in Imaris (Bitplane, Switzerland). We quantified the biomass of the two tagged populations with the open source software FIJI (Loci) and the plug-in Voxel_Counter (NIH).

### Biofilm selection experiment.

To determine potential fitness of PSL production, we performed a selection experiment over 6 rounds of growth, with four different treatment regimens and relatively low and relatively high relatedness (Lr and Hr, respectively) (Fig. S5). The Hr treatment started with three 3-ml aliquots of subpopulations of diluted logarithmic-phase PSL^+^ cells and PSL^−^ cells (*n* = 6). In contrast, the Lr treatment started with six subpopulations of a 3-ml 1:1 mix of PSL^+^ and PSL^−^ cells (*n* = 6). We grew cultures at 37°C with shaking at 200 rpm for 24 h in the presence/absence of plastic beads. We recovered biofilm cells as detailed previously and pooled the cells prior to serial dilution and plating. We recovered 1 ml of unattached cells from the liquid medium without plastic beads and subjected the cells to the same treatment (omitting the three wash stages). We determined the PSL^+^:PSL^−^ cell ratio for each treatment regimen phenotypically; PSL^+^ and PSL^−^ cells have rugose and smooth colony morphologies, respectively. For Hr treatments, we then randomly selected six clones to inoculate 3 ml of M9 citrate and progress to the next round, whereas for LR treatments, we randomly took a sweep of multiple colonies. We grew cultures at 37°C with shaking at 200 rpm for 24 h in the presence/absence of plastic beads, with an additional round of selection. We repeated this experiment six times, with six selection lines per treatment regimen.

### Mathematical formula and statistics.

We determined the relative fitness (*w*) of the PSL^−^ strain by using the following formula: *w* = *x*_2_(1 − *x*_1_)/x_1_(1 − *x*_2_), where *x*_1_ is the starting mutant proportion of the population and *x*_2_ is the end mutant proportion (36). All error bars denote standard errors of the means. All statistical tests, including unpaired *t* tests, linear regression analyses, and one-way and two-way analyses of variance were performed on the VassarStats website (http://vassarstats.net/).
