# Supplementary figures and images for: The Pseudomonas aeruginosa PSL Polysaccharide Is a Social but Noncheatable Trait in Biofilms
Source: mBio. 2017 Jun 20;8(3):e00374-17. doi: 10.1128/mBio.00374-17 (PMC5478892; doi:10.1128/mBio.00374-17)

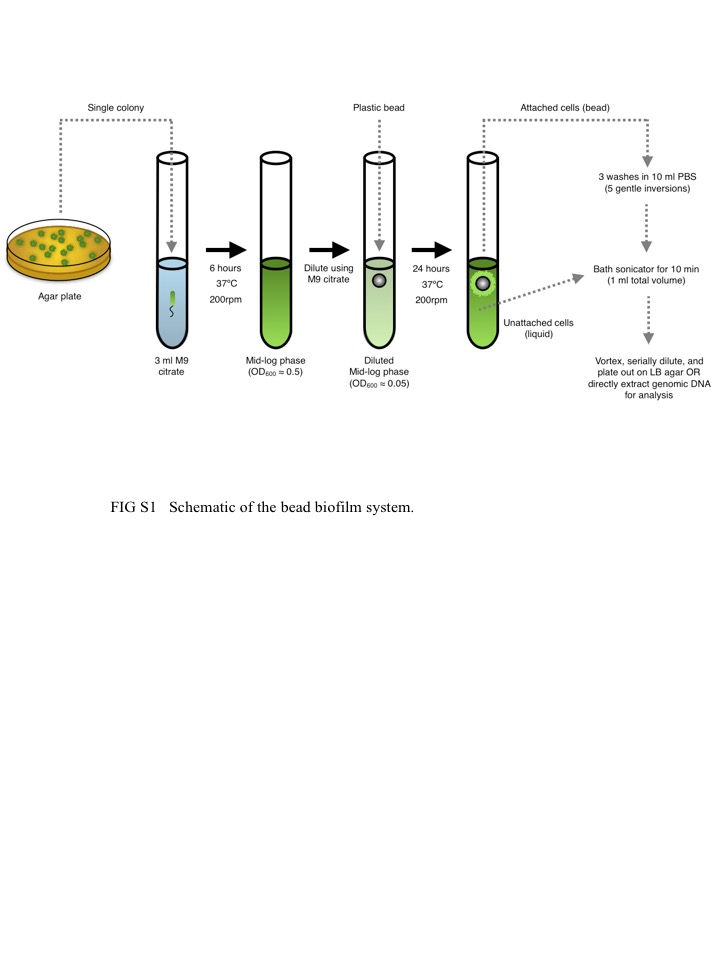

Supplement: FIG S1 [file mbo003173346sf1.jpg]

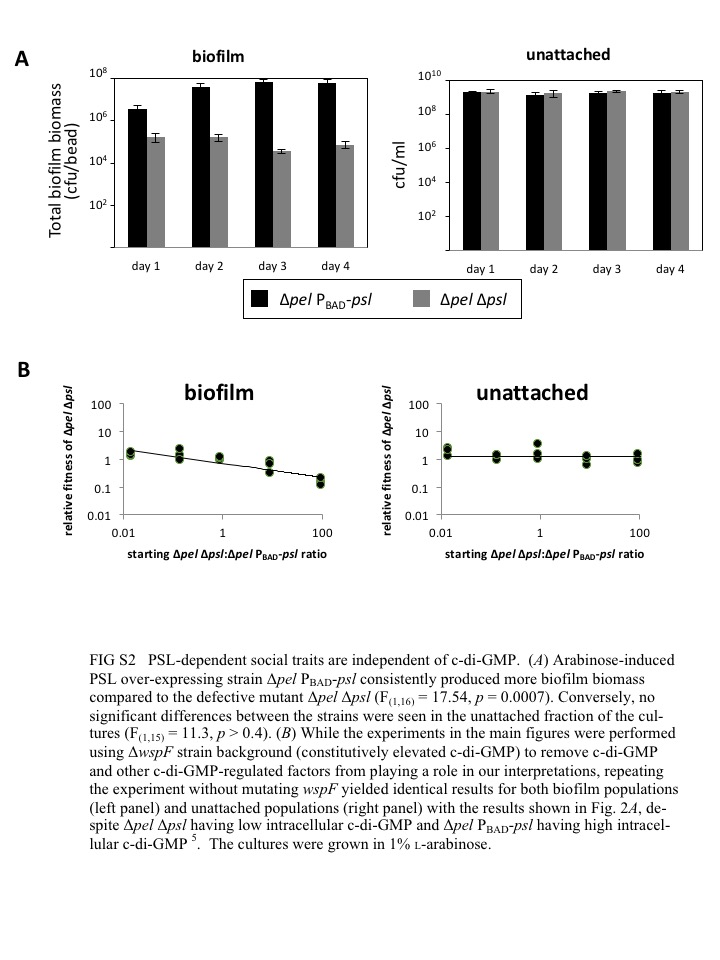

Supplement: FIG S2 [file mbo003173346sf2.jpg]

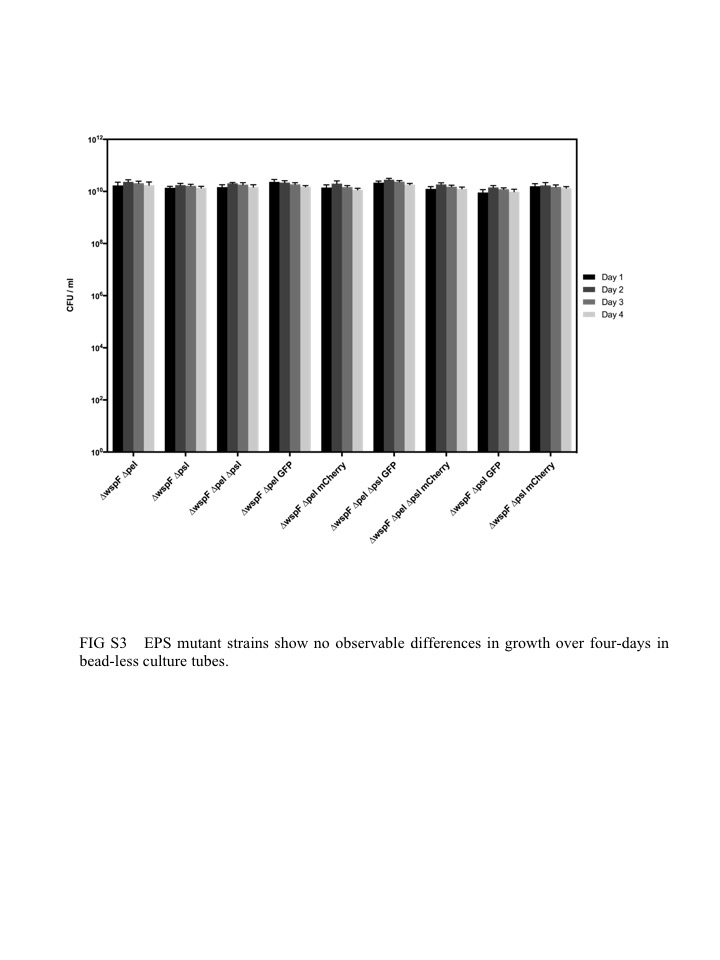

Supplement: FIG S3 [file mbo003173346sf3.jpg]

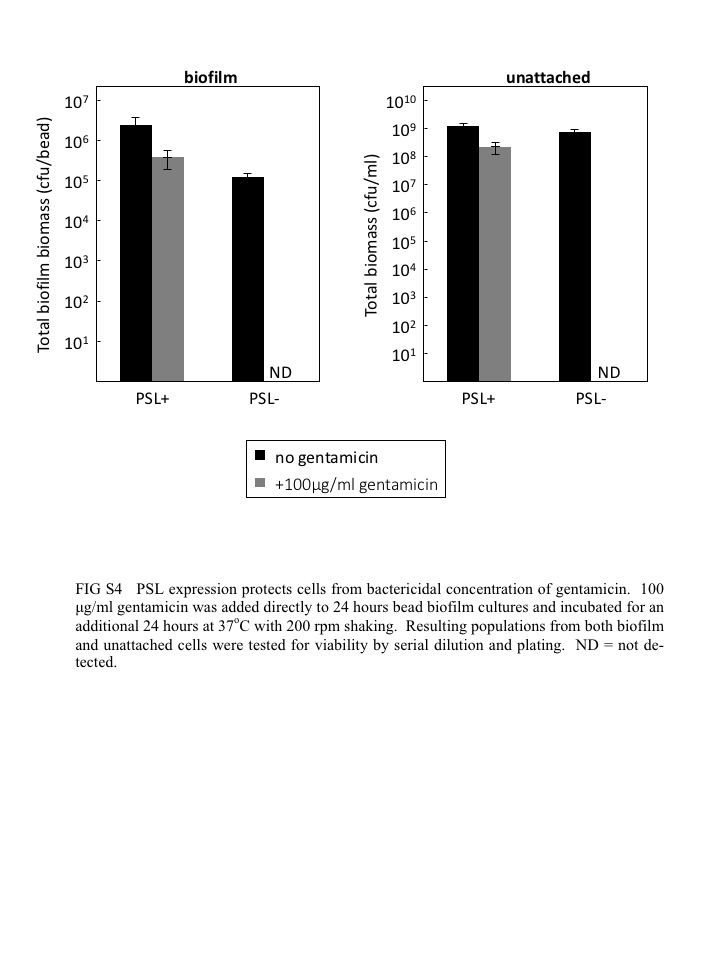

Supplement: FIG S4 [file mbo003173346sf4.jpg]

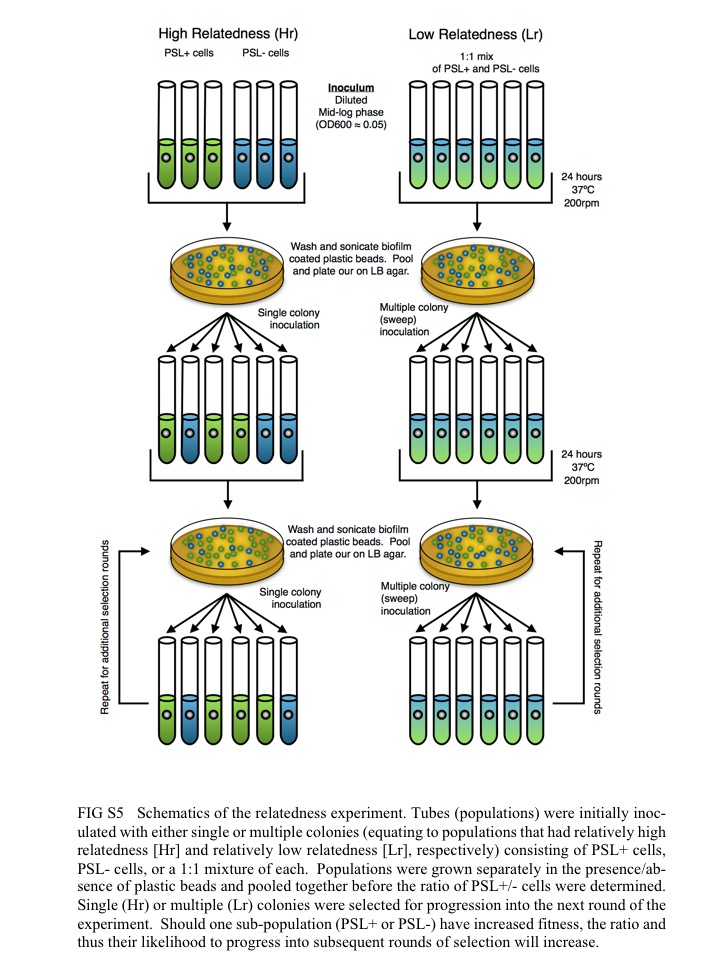

Supplement: FIG S5 [file mbo003173346sf5.jpg]
