# Supplementary material for: The Pseudomonas aeruginosa PSL Polysaccharide Is a Social but Noncheatable Trait in Biofilms
Source: mBio. 2017 Jun 20;8(3):e00374-17. doi: 10.1128/mBio.00374-17 (PMC5478892; doi:10.1128/mBio.00374-17)
Supplement: FIG S6 [file mbo003173346sf6.pdf]

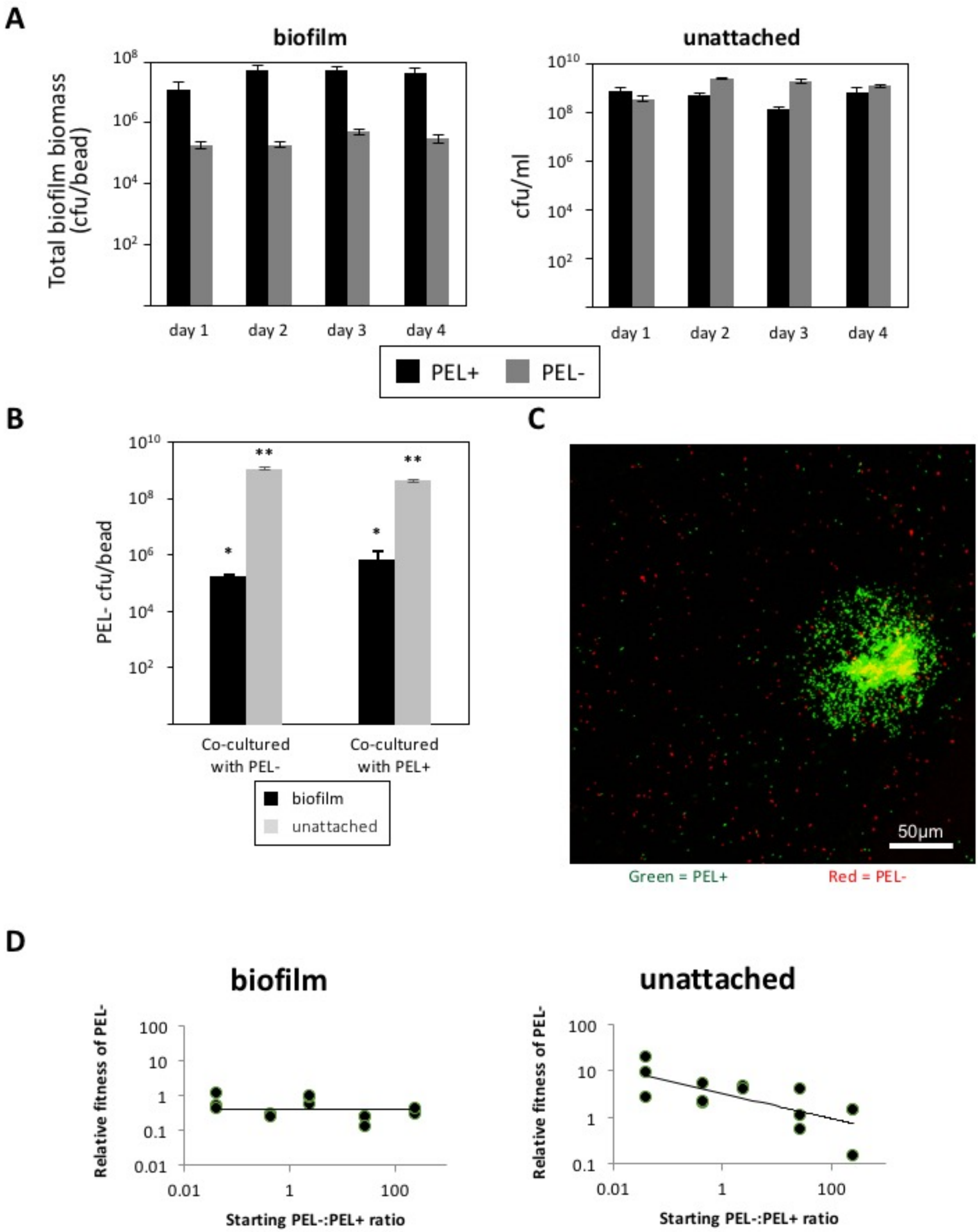
A
biofilm
Total biofilm biomass (cfu/bead)
day 1
day 2
day 3
day 4
unattached
cfu/ml
PEL+
PEL-
B
PEL- cfu/bead
Co-cultured with PEL-
Co-cultured with PEL+
biofilm
unattached
C
50μm
Green = PEL+
Red = PEL-
D
biofilm
unattached
Relative fitness of PEL-
Starting PEL-:PEL+ ratio

E

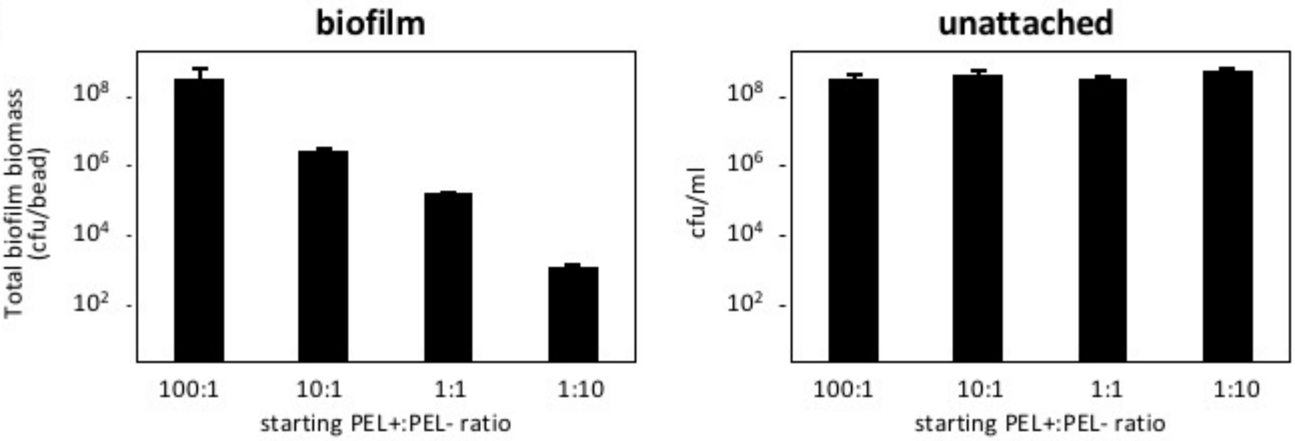


FIG S6 PEL polysaccharides are private goods. (*A*) Similar to PSL (Fig. 1a), PEL- strain is significantly defective in biofilm formation compared to PEL+ ($F_{(1,24)}$ = 27.3, $p$ < 0.0001, $n$ = 4), but no major differences are seen between their growths in the unattached fractions ($F_{(1,24)}$ = 46.56, $p$ < 0.0001, $n$ = 4. (*B*) Unlike PSL, co-cultures of PEL- strains with PEL+ strains do not increase the amount of PEL- strains in neither biofilm nor unattached populations, signifying the unavailability of PEL polysaccharides to non-producing cells. * and ** $p$ > 0.02. (*C*) Confocal micrograph image of surface attached populations of PEL-/PEL+ 1:1 co-cultures. PEL- cells (red) do not co-aggregate with PEL+ cells (green). (*D*) There is no frequency-dependent fitness changes for PEL-/PEL+ co-cultured biofilm ($t$(13) = -0.802, $p$ = 0.4371), and the relative fitness is consistently slightly below 1, indicating a steady disadvantage of not expressing PEL in the biofilm. There is a frequency-dependent fitness changes in the unattached population ($t$(13) = -1.435, $p$ = 0.175), perhaps caused by complex regulatory system of PEL and the involvement of quorum sensing [8]. (*E*) Due to PEL not socially affecting co-culture communities, there is a steady decline of biofilm biomass as PEL+ becomes rarer ($F_{(1,8)}$ = 1, $p$ = 0.441099, $n$ = 3), but no change is seen in the maximum cell numbers in the unattached population ($F_{(1,8)}$ = 1.46, $p$ = 0.296592, $n$ = 3) regardless of the starting ratios of the strains.
