# Supplementary material for: The Pseudomonas aeruginosa PSL Polysaccharide Is a Social but Noncheatable Trait in Biofilms
Source: mBio. 2017 Jun 20;8(3):e00374-17. doi: 10.1128/mBio.00374-17 (PMC5478892; doi:10.1128/mBio.00374-17)
Supplement: TABLE S1 [file mbo003173346st1.docx]

**Table S1.** Bacterial strains and plasmids used in this study.

| Strains/plasmids | Description | Source/reference |
| --- | --- | --- |
| ***Pseudomonas aeruginosa*** |  |  |
| PAO1 | wild type sequenced strain | (1) |
| Δ*pel* | defective for PEL production | (2) |
| Δ*pel* GFP | Δ*pel* expressing chromosomally labelled constitutive GFP | This study |
| Δ*pel* mCherry | Δ*pel* expressing chromosomally labelled constitutive mCherry | This study |
| Δ*psl* | defective for PSL production | (3) |
| Δ*psl* GFP | Δ*psl* expressing chromosomally labelled constitutive GFP | This study |
| Δ*psl* mCherry | Δ*psl* expressing chromosomally labelled constitutive mCherry | This study |
| Δ*wspF* Δ*pel* | constitutively elevated intracellular c-di-GMP; PEL defective | (4) |
| Δ*wspF* Δ*pel* GFP | Δ*wspF* Δ*pel* expressing chromosomally labelled constitutive GFP | This study |
| Δ*wspF* Δ*pel* mCherry | Δ*wspF* Δ*pel* expressing chromosomally labelled constitutive mCherry | This study |
| Δ*wspF* Δ*pel* Δ*psl* | constitutively elevated intracellular c-di-GMP; defective in both PEL and PSL | (2) |
| Δ*wspF* Δ*pel* Δ*psl* GFP | Δ*wspF* Δ*pel* Δ*psl* expressing chromosomally labelled constitutive GFP | This study |
| Δ*wspF* Δ*pel* Δ*psl* mCherry | Δ*wspF* Δ*pel* Δ*psl* expressing chromosomally labelled constitutive mCherry | This study |
| Δ*wspF* Δ*psl* | constitutively elevated intracellular c-di-GMP; PSL defective | (4) |
| Δ*wspF* Δ*psl* GFP | Δ*wspF* Δ*psl* expressing chromosomally labelled constitutive GFP | This study |
| Δ*wspF* Δ*psl* mCherry | Δ*wspF* Δ*psl* expressing chromosomally labelled constitutive mCherry | This study |
| Δ*pel* P_BAD_-*psl* | Arabinose-inducible PSL over-expressing strain (Δ*pel* background) | (5) |
| Δ*pel* P_BAD_-*psl* GFP | Δ*pel* P_BAD_-*psl* expressing chromosomally labelled constitutive GFP | This study |
| Δ*pel* P_BAD_-*psl* mCherry | Δ*pel* P_BAD_-*psl* expressing chromosomally labelled constitutive mCherry | This study |
| Δ*pel* Δ*psl* | defective in both PEL and PSL | (2) |
| Δ*pel* Δ*psl* GFP | Δ*pel* Δ*psl* expressing chromosomally labelled constitutive GFP | This study |
| Δ*pel* Δ*psl* mCherry | Δ*pel* Δ*psl* expressing chromosomally labelled constitutive mCherry | This study |
| ***Escherichia coli*** |  |  |
| DH5α | cloning strain F′/*endA1 hsdR17*(r_k_-m_k_+) *glnV44 thi-1 recA1* *gyrA* (NalR) *relA1* Δ*(lacIZYA-argF)U169 deoR* (φ80*dlac*Δ*(lacZ)M15*) | Gibco/BRL |
| XL-10 Gold | cloning strain *endA1* *glnV44* *recA1 thi-1 gyrA96 relA1 lac Hte* Δ*(mcrCB-hsdSMR-mrr)173 tet^R^* F′*[proAB lacI^q^ZΔM15 Tn10(Tet^R^ Amy Tn5(Kan^R^))]* | Stratagene |
| **Plasmids** |  |  |
| pFLP2 | FLP recombinase expressing plasmid  Amp/CarbR | (6) |
| pTNS3 | Tn7 transposase helper plasmid | (7) |
| pUC18-mini-Tn7T2-PA1/04/03::*gfp* | Suicide vector carrying Tn7 transposable constitutive GFP construct | This study |
| pUC18-mini-Tn7T2-PA1/04/03::*mcherry* | Suicide vector carrying Tn7 transposable constitutive mCherry construct | This study |
