# Supplementary material for: The Pseudomonas aeruginosa PSL Polysaccharide Is a Social but Noncheatable Trait in Biofilms
Source: mBio. 2017 Jun 20;8(3):e00374-17. doi: 10.1128/mBio.00374-17 (PMC5478892; doi:10.1128/mBio.00374-17)
Supplement: TABLE S2 [file mbo003173346st2.docx]

**Table S2.** Real-time PCR oligonucleotide primers used in this study.

| Oligonucleotide primer | Sequence (5' to 3') |
| --- | --- |
| *gfp* RT for1 | CGAAAGATCCCAACGAAAAGAG |
| *gfp* RT rev1 | TCCCAGCAGCTGTTACAAACTC |
| *mCherry* RT for1 | TTGGACATCACCTCCCACAA |
| *mCherry* RT rev1 | TCGGCGCGTTCGTACTGT |
